# Targeting type H vessels with bioactive metabolites from traditional Chinese botanical drugs: a therapeutic strategy for skeletal disorders

**DOI:** 10.3389/fphar.2025.1693131

**Published:** 2025-10-24

**Authors:** Shengping Tang, Jinkao Li, Zhuqing Dong, Yangjie Cai, Jianwei Hu, Xiaofei Ding, Shijie Liao

**Affiliations:** ^1^ Department of Orthopedic Trauma and Hand Surgery, The First Affiliated Hospital of Guangxi Medical University, Nanning, China; ^2^ Collaborative Innovation Centre of Regenerative Medicine and Medical Bioresource Development and Application Co-constructed by the Province and Ministry, Guangxi Medical University, Nanning, China; ^3^ The Second Clinical Medical College of Guangxi Medical University, Nanning, China; ^4^ Guangxi Key Laboratory of Regenerative Medicine, Orthopaedic Department, The First Affiliated Hospital of Guangxi Medical University, Nanning, China

**Keywords:** bioactive metabolites, type H vessels, osteoporosis, osteoarthritis, bone defects, fracture, osteonecrosis of the femoral head

## Abstract

Type H vessels are a specialized subtype of bone capillaries, first identified in 2014, characterized by high co-expression of CD31 and Endomucin. These vessels play a key regulatory role in bone development, repair, and remodeling through angiogenesis–osteogenesis coupling, which is essential for maintaining skeletal homeostasis. Type H vessels are abundant in the bones of young individuals but gradually decline with age, and their dysregulation is closely associated with skeletal disorders, including osteoporosis, osteoarthritis, bone defects, fractures, and osteonecrosis of the femoral head. Previous studies have identified the molecular mechanisms underlying the regulation of type H vessels, and recent investigations have examined pharmacological strategies to modulate these pathways. Among these, bioactive metabolites derived from traditional Chinese botanical drugs have attracted attention for their ability to regulate type H vessel formation and improve skeletal health. This review summarizes the molecular mechanisms by which these bioactive metabolites target type H vessels, highlighting their therapeutic potential in skeletal disorders and suggesting that modulation of type H vessel formation represents a promising strategy for intervention. Future studies are needed to further clarify the mechanisms of action of these metabolites and to assess their safety and clinical efficacy for translation into human therapy.

## 1 Introduction

Throughout the human lifespan, bone development, growth, repair, and remodeling are critical physiological processes that play a decisive role in maintaining skeletal health and normal function. The proper execution of these processes relies on the coordinated actions of multiple cell types, among which intricate interactions exist between the bone vascular system and cells such as osteoblasts and osteoclasts (Salhotra et al., 2020). The bone vascular system is vital for bone homeostasis and regeneration. In addition to its fundamental functions in nutrient delivery and metabolic waste removal, vascular endothelial cells secrete various bioactive signaling molecules that directly or indirectly regulate the proliferation, differentiation, and function of bone cells, thereby coordinating angiogenesis and osteogenesis (Grosso et al., 2017; Tuckermann and Adams, 2021). Osteoblasts, as the primary functional cells responsible for bone formation through bone matrix synthesis and mineralization, require a sufficient nutrient supply for normal activity and can act in a paracrine manner by releasing signaling molecules that stimulate adjacent vascular endothelial cells to promote vascularization, thereby enhancing bone matrix synthesis and mineralization to facilitate osteogenesis (Hu and Olsen, 2016; Chen K. et al., 2021). Osteoclasts primarily mediate bone resorption and, through balanced interactions with osteoblasts, maintain the dynamic equilibrium between bone resorption and formation. Recent studies have revealed that, in addition to classical bone-resorbing osteoclasts, a distinct population of vessel-associated osteoclasts (VAOs) exists at the bone/cartilage junction in the proximal metaphysis (Romeo et al., 2019). Bone vascular endothelial cells can support VAOs through the RANKL/RANK signaling pathway. VAOs promote the anastomosis of bone capillaries, restrict excessive vascular invasion into cartilage, and help maintain normal bone morphology and growth direction. Such intercellular regulatory mechanisms within the bone microenvironment are crucial for sustaining normal bone metabolism and structural stability (Hu and Olsen, 2016; Stegen and Carmeliet, 2024). Moreover, research has revealed multiple vascular subtypes in bone, among which type H vessels were the first to be identified and have become a major focus due to their critical roles in bone development, growth, and repair (Kusumbe et al., 2014). Type H vessels are a specialized capillary subtype in bone. Temporally, their formation precedes osteogenesis, providing a prerequisite for osteoblast-mediated bone formation and mineralization; Spatially, type H vessels are densely surrounded by Runx2^+^ osteoprogenitors and Col1α1^+^ osteoblasts while simultaneously expressing PDGF-α and TGF-β1 to drive osteoprogenitor differentiation into osteoblasts and osteocytes, thereby enhancing bone formation (Kusumbe et al., 2014). This spatiotemporal interdependence between angiogenesis and osteogenesis is termed “angiogenesis-osteogenesis coupling” (Grosso et al., 2017; Peng et al., 2020; Li S. et al., 2025).

Bioactive metabolites are natural small molecules produced through the metabolic processes of various organisms, including edible plants, animals, and microorganisms, which can modulate physiological and pathological functions and hold significant potential for promoting human health (Kussmann et al., 2023). Plant-derived bioactive metabolites, primarily secondary metabolites such as flavonoids, alkaloids, and phenolic compounds, are typically characterized by low toxicity, high biocompatibility, and diverse pharmacological activities. Many of these metabolites are enriched in traditional Chinese botanical drugs historically used to treat skeletal disorders. Botanical drugs such as Duzhong (Eucommia ulmoides), Gusuibu (Rhizoma Drynariae), and Yinyanghuo (Epimedium sagittatum) have been traditionally used in traditional Chinese medicine (TCM) to “strengthen bones” and “promote fracture healing,” reflecting a long-standing ethnopharmacological heritage. Critically, modern research is now elucidating the molecular mechanisms of these classical TCM formulas, revealing that their osteoprotective effects involve the regulation of type H vessels. For example, Zhuang-Gu-Fang, composed of Epimedium sagittatum, Astragalus membranaceus, Dioscorea opposita, Eucommia ulmoides, and Salvia miltiorrhiza, alleviates diabetic osteoporosis by promoting type H vessel formation *via* the Notch1/Noggin/VEGF pathway, enhancing angiogenic–osteogenic coupling and bone regeneration (Jin et al., 2024). Du-Zhong-Wan, consisting of Eucommia ulmoides and Dipsacus asper, facilitates type H vessel formation in osteoporotic fractures by upregulating the osteoblast-derived coupling factor SLIT3 (Tian et al., 2022). Furthermore, Modified Qing’e Pills, composed of Eucommia ulmoides, Fructus Psoraleae, Semen Juglandis, and Allium sativum, prevent bone loss by enhancing type H vessel formation through the HIF-1α/VEGF pathway and suppressing RAAS activation (Lu et al., 2022), while Gushukang, consisting of Herba Epimedii, Rehmannia glutinosa, and Rhizoma Drynariae, increases bone mass by promoting type H vessel formation and osteogenesis *via* HIF-1α upregulation (Li et al., 2020). These ethnopharmacological practices provide important guidance for modern pharmacological research, linking traditional therapeutic concepts to molecular evidence.

Notably, TCM formulas consist of multiple botanical drugs, and the specific bioactive metabolites responsible for their therapeutic effects remain largely unelucidated. Promisingly, recent studies have begun to identify plant-derived metabolites that can regulate type H vessel formation. These metabolites act through multiple mechanisms, which include not only enhancing osteoblast activity, promoting bone formation, and inhibiting excessive bone resorption by osteoclasts, but also regulating type H vessel formation to modulate bone remodeling and treat skeletal diseases (Ji et al., 2018; Song et al., 2020; Wang et al., 2021; Li et al., 2022). Accumulating evidence suggests that bioactive metabolites confer therapeutic benefits in osteoporosis, osteoarthritis, fracture repair, osteonecrosis of the femoral head, and other related skeletal disorders through these mechanisms. In this context, we conducted a systematic literature search in PubMed and Web of Science, focusing primarily on peer-reviewed English articles published within the past 10 years, while also including some valuable earlier works. The search strategy employed combinations of keywords including “type H vessel,” “H-type vessel,” “osteoporosis,” “osteoarthritis,” “fracture,” “bone defect,” “osteonecrosis of the femoral head,” and “skeletal disorders,” together with terms related to natural products and ethnopharmacology, such as “natural product,” “traditional Chinese medicine,” “herbal formula,” “botanical drug,” and “natural small-molecule compounds.” This review summarizes the role of type H vessels in skeletal disease pathogenesis and progression, with particular emphasis on recent advances in understanding the molecular mechanisms by which bioactive metabolites from traditional Chinese botanical drugs treat bone diseases through type H vessel regulation, providing theoretical foundations for clinical application of these metabolites and the development of novel therapeutic strategies.

## 2 Type H vessels and their regulatory factors in bone

In 2014, Kusumbe et al. discovered a specialized microvascular subtype in murine bone, defined by high co-expression of CD31 and Endomucin (Emcn) and thus named type H vessels (Kusumbe et al., 2014). In contrast, vessels exhibiting low co-expression of these markers were classified as type L vessels, with significant morphological and functional differences existing between these two vascular subtypes. Type H vessels predominantly localize to metaphyseal and endosteal regions with active bone growth, displaying columnar distribution patterns that form arch-like structures adjacent to the growth plate, while type L vessels are mainly distributed in the diaphysis with reticular networks. During skeletal development, type H vessel abundance demonstrates dynamic changes: they are highly prevalent during juvenile and adolescent stages to support rapid bone growth but gradually decline with aging (Kusumbe et al., 2014). Subsequent studies confirmed the existence of type H vessels in human bone, showing similar age-dependent reduction, suggesting their potential as specific biomarkers for bone mass (Wang L. et al., 2017; Zhu et al., 2019; Gao et al., 2020). At the termination of growth, type H vessels undergo the phenotypic transition to type L vessels, a process potentially regulated by mechanical loading. When mechanical stress reaches a threshold, the mechanosensor protein PIEZO1 is significantly upregulated, stimulating massive secretion of phosphorylated dentin matrix protein 1 (DMP1), which drives the conversion of metaphyseal type H to type L vessels, thereby limiting bone elongation (Dzamukova et al., 2022). Furthermore, recent studies have identified other vascular subtypes in bone, including type E, S, and R vessels, which collectively maintain bone homeostasis (Table 1). Type E vessels are primarily located in the metaphysis of long bones during early embryonic development and exhibit higher CD31 expression than type H vessels, with the capacity to promote osteogenic differentiation of Osx^+^ osteoprogenitors surrounding bone vasculature in both embryonic and postnatal stages, while maintaining strong potential to differentiate into type H vessels, which continue to mediate bone growth and development (Langen et al., 2017). Type S vessels are named after their discovery in the secondary ossification center of mouse long bones, and they exhibit high expression of Ly6a, Ly6c1, and COLA1, with the ability to secrete type I collagen, playing an important role in promoting epiphyseal growth and development and in maintaining the homeostasis of the hematopoietic stem cell microenvironment (Iga et al., 2023). Type R vessels represent a capillary subtype characterized by high expression of Emcn and Cav1 but lacking Flt4 expression, predominantly localized around trabeculae in adult murine bone, with their abundance increasing in aged mice and positively correlating with improved bone quality, suggesting potential therapeutic relevance for osteoporosis treatment (Mohanakrishnan et al., 2024).

**TABLE 1:** Identified vascular subtypes in bone.

Vascular subtype	Growth characteristics	Anatomical features	Molecular markers	Primary functions	References
Type H vessels	Abundant during juvenile growth, progressively declines with aging	Metaphysis and endosteum of long bones; columnar distribution, forming arch structures near the growth plate; also observed in vertebrae and mandibular condyle	High CD31, High Emcn	Promotes osteoprogenitor differentiation into osteoblasts, strongly couples angiogenesis-osteogenesis	Kusumbe et al. (2014)
Type E vessels	Highly abundant during embryonic and early postnatal stages	Metaphysis and endosteum of long bones	Higher CD31 (> type H), Lower Emcn (< type H), also expresses Esm1, Kitl, Unc5b, Bcam, Cav1, Apln	Exhibits a stronger association with Osx^+^ osteoprogenitors than type H vessels. Differentiates into type H vessels during development	Langen et al. (2017)
Type S vessels	Emerges at postnatal day 7 (P7) in mice, increases during P7-P28	Secondary ossification centers of long bones; tree-like/dendritic morphology	High Ly6a, also expresses Ly6c1, Col1a1, Col1a2	Facilitates secondary ossification center formation and bone mineralization	Iga et al. (2023)
Type R vessels	Gradually increases from the juvenile to the aged stages in mice	Peritrabecular localization	High Emcn, High Cav1, Low CD31, Lack of Flt4 and VEGFR3	Potentially regulates skeletal maturation and remodeling, promotes osteogenesis with anti-osteoporosis potential	Mohanakrishnan et al. (2024)

Since its discovery, many scholars have continuously studied the molecular mechanisms regulating type H vessels. It is now known that their formation and function are finely regulated by various molecules. Platelet-derived growth factor-BB (PDGF-BB) is primarily secreted by osteoclast precursor cells (OPCs), which can specifically bind to PDGFRβ to exert its effects, studies have shown that PDGF-BB can significantly promote type H vessel formation, thus driving bone formation (Xie et al., 2014; Cao et al., 2023). Slit guidance ligand 3 (SLIT3) was initially discovered in the central nervous system, where it mediates axon guidance through interactions with the Robo family. Interestingly, in the skeletal system, SLIT3 also mediates endothelial cell migration and chemotaxis, accelerates the formation of endothelial vascular networks, and treatment with SLIT3 can promote type H vessel formation, increase bone formation, and facilitate bone regeneration (Xu et al., 2018; Li J. et al., 2023). Hypoxia-inducible factor-1α (HIF-1α) is upregulated under hypoxic conditions and can enhance the expression and activity of vascular endothelial growth factor-A (VEGF-A), promoting the generation of type H vessels (Kusumbe et al., 2014). Notch signaling is regulated by blood flow, with vessel diameter and flow velocity being key modulatory factors in endothelial cell Notch signaling that influence bone angiogenesis, and studies have shown that reactivation of Notch signaling in endothelial cells of aged mice can restore type H vessel abundance and increase bone mass (Ramasamy et al., 2016). In osteoarthritis, TGF-β plays an important role in enhancing chondrocyte regeneration and maintaining cartilage homeostasis, but studies have found that TGF-β can also drive the pathological formation of type H vessels, leading to the development of osteoarthritis, while inhibition of TGF-β expression can alleviate the disease, indicating the dual nature of its function (Cui et al., 2016; Kim et al., 2022; Liu Y. et al., 2023). Epidermal growth factor-like domain 6 (EGFL6) is specifically secreted by osteoblasts, which promote osteogenesis by inducing the BMP/Smad pathway through intracellular secretion of EGFL6, while paracrine secretion of EGFL6 acts on adjacent vascular endothelial cells to promote the formation of type H vessels (Chen K. et al., 2021). EGFL6 can also promote the formation of type H vessels through the Wnt/β-catenin pathway in a dose-dependent manner (Shen et al., 2021). Vascular endothelial growth factor-A (VEGF-A), secreted by both osteoblasts and chondrocytes, serves as another critical inducer of type H vessel generation that facilitates bone repair processes (Bai et al., 2021; Liu X. et al., 2023). Importantly, the expression of VEGF-A is regulated by factors such as HIF-1α, PDGF-BB, and EGFL6, indicating that the formation of type H vessels is jointly regulated by multiple factors and signaling pathways within the bone microenvironment. Moreover, as a type of non-coding RNA, miRNA can also participate in the regulation of type H vessel formation by binding to the 3′UTR of target mRNAs (Zhang D. et al., 2024). Studies have found that miR-136-3p can suppress the expression of the PTEN gene, thereby rescuing impaired type H vessel formation and bone density loss caused by long-term excessive alcohol consumption (Chen et al., 2020). Prenatal caffeine exposure upregulates miR-375, which inhibits connective tissue growth factor expression in growth plates, leading to reduced type H vessel abundance and impaired fetal long bone development (He et al., 2021). During aging, increased expression of miR-188-3p in endothelial cells negatively regulates type H vessels through suppression of integrin β3 (He et al., 2022). Currently, an increasing number of studies have found that bioactive metabolites can regulate type H vessel formation through the aforementioned or additional molecular mechanisms, thereby playing a potential role in the treatment of type H vessel-related skeletal disorders.

## 3 Regulation of type H vessel angiogenesis by bioactive metabolites in skeletal disorders

The pathogenesis of skeletal disorders is closely associated with dysregulated intraosseous angiogenesis (Tuckermann and Adams, 2021). As the key functional unit in “angiogenesis-osteogenesis coupling”, type H vessels critically influence bone repair, remodeling, and homeostasis maintenance. Excessive abundance and insufficiency of type H vessels are important pathogenic factors driving the progression of diseases such as osteoporosis, osteoarthritis, bone defects, fractures, and osteonecrosis of the femoral head (Peng et al., 2020; Liu X. et al., 2023; Zhang et al., 2023). Emerging studies have shown that bioactive metabolites derived from traditional botanical drugs can modulate disease progression by targeting type H vessel formation, particularly through the regulation of critical signaling pathways mediated by PDGF-BB, VEGF-A, and additional angiogenic regulators. These metabolites exert their pharmacological effects through a cascade of “modulating type H vessel generation - regulating bone microenvironment - reversing disease phenotypes”. The following sections will explore the regulatory network of type H vessels in conditions such as osteoporosis, osteoarthritis, bone defects, fractures, and osteonecrosis of the femoral head, providing a theoretical framework for a deeper understanding of the mechanisms by which bioactive metabolites exert their effects.

### 3.1 Osteoporosis

Osteoporosis (OP), a prevalent systemic bone disorder, is characterized by decreased bone mineral density, compromised bone quality, and deteriorated microarchitecture, collectively leading to enhanced bone fragility. This condition can be triggered by multiple factors, including aging and estrogen deficiency (Ye et al., 2025). According to the Seventh National Population Census released by the National Bureau of Statistics of China in May 2021, the population aged 60 and above in China is 264 million (accounting for 18.7% of the total population), and the population aged 65 and above exceeds 190 million (accounting for 13.5%). Epidemiological surveys indicate that the prevalence of OP among people aged 50 and above in China is 19.2%, including 32.1% in women and 6.9% in men, and 32.0% among those aged 65 and above, with the rate in women reaching as high as 51.6%. Based on these data, it is estimated that there are approximately 90 million OP patients in China, of whom about 70 million are women (Chinese Society of Osteoporosis and Bone Mineral Research, 2023). With the intensifying aging of the population in China, the prevalence of OP is rapidly increasing and has become a serious public health issue. The pathogenesis and progression of OP are closely associated with age-related depletion of type H vessels (Ding et al., 2020). A study of 134 Chinese women demonstrated a positive correlation between type H vessel density and femoral neck/total hip bone mineral density (BMD), with OP patients exhibiting significantly reduced type H vessel proportions, suggesting its potential as a bone mass biomarker in OP (Zhu et al., 2019). While bisphosphonates remain the most widely prescribed anti-osteoporotic drugs by inhibiting osteoclast-mediated bone resorption, their use is limited by adverse effects, including renal impairment, osteonecrosis of the jaw, and atypical femoral fractures (Silverman et al., 2018; Reid et al., 2020; Ferreira Jr et al., 2021). In contrast, bioactive metabolites have garnered increasing attention due to their favorable safety profiles and therapeutic efficacy. Accumulating evidence indicates these metabolites may treat OP by modulating type H vessel formation, positioning them as promising next-generation therapeutic strategies.

Emerging research has identified several bioactive metabolites that exert anti-osteoporotic effects by stimulating PDGF-BB secretion from OPCs to modulate type H vessel formation. Harmine, a naturally occurring tricyclic β-carboline alkaloid that can be extracted from *Peganum harmala* L. [Nitrariaceae], demonstrates diverse pharmacological properties, including anti-inflammatory and anti-tumor activities (Zhang L. et al., 2020; Hu et al., 2024). Harmine can promote OPCs to secrete PDGF-BB and simultaneously increase the expression level of VEGF in the bone marrow cavity, thereby inducing type H vessel formation (Huang et al., 2018). It exerts a bone-protective effect in ovariectomized (OVX) mouse models, reducing bone loss. However, direct treatment with Harmine inhibits endothelial angiogenesis, suggesting that its pharmacological effect of promoting bone angiogenesis may be mediated through the above-mentioned indirect mechanism. Given harmine’s potential central nervous system toxicity, the authors formulated it as an oral oil-in-water emulsion. In this form, OVX mice showed no signs of neurotoxicity, while bone-protective effects were preserved, offering a safer strategy for osteoporosis therapy.

Nuciferine (NCF), an aporphine-type alkaloid isolated from the leaves of *Nelumbo nucifera* Gaertn. [Nelumbonaceae], possesses notable anti-inflammatory and anti-obesity capacities (Wan et al., 2022; Li et al., 2024). NCF also exhibits anti-osteoporotic effects. Mechanistically, NCF inhibits the fusion of Trap^+^ OPCs into multinucleated osteoclasts and promotes the secretion of PDGF-BB from Trap^+^ OPCs to induce type H vessel formation, and the inhibition of the MAPK/NF-κB/c-Fos/NFATc1 signaling pathways may be involved in this process (Song et al., 2020). However, the 40 mg/kg dose of NCF used in their study may not be sufficient to fully prevent osteoporosis in OVX mice, and its low absorption and rapid clearance limit efficacy, indicating that further optimization of dosage and delivery strategies is needed.

Aucubin is an iridoid glycoside metabolite extracted from the leaves of Aucuba japonica Thunb. [Garryaceae] and Eucommia ulmoides Oliv. [Eucommiaceae], which exhibits multiple pharmacological effects, including anti-inflammatory and antioxidant properties (Shen et al., 2019; Wang et al., 2020b), as well as bone-protective activity. Through analogous mechanisms involving MAPK/NF-κB pathway inhibition and subsequent PDGF-BB upregulation in OPCs, aucubin promotes type H vessel formation to combat osteoporosis (Li et al., 2022). However, the study relied on intraperitoneal injection at 5 mg/kg, which is not an ideal administration route for a chronic condition like osteoporosis; in addition, the oral bioavailability and pharmacokinetic profile of aucubin remain unclear, limiting its translational potential.

Acacetin is a flavonoid metabolite extracted from plants such as *Vachellia farnesiana* (L.) Wight & Arn. [Fabaceae], among others, and it exhibits notable anti-inflammatory, anti-tumor, and anti-osteoporotic activities (Wang et al., 2020a; Wang S. et al., 2020; Sun et al., 2022; Bu et al., 2024). Acacetin effectively inhibits osteoclast differentiation from bone marrow-derived macrophages (BMMs). While direct Acacetin treatment suppresses the angiogenic capacity of endothelial progenitor cells (EPCs), its modulation of OPCs indirectly enhances EPC proliferation and migration, thereby promoting angiogenesis. Mechanistically, Acacetin inhibits Akt/GSK3β and NF-κB signaling pathways, downregulates osteoclastogenic transcription factors (e.g., NFATc1), and simultaneously promotes PDGF-BB secretion from OPCs to stimulate type H vessel formation, ultimately ameliorating bone loss in OVX mice (Lin et al., 2022). Future studies should explore the optimal dose and pharmacokinetic profile of Acacetin to support its clinical translation.

In addition to the PDGF-BB–dependent metabolites described above, other bioactive metabolites can promote type H vessel formation and exert anti-osteoporotic effects *via* VEGF-mediated mechanisms. Diabetic osteoporosis is a metabolic disorder characterized by hyperglycemia-induced abnormalities in bone microarchitecture and reduced bone mineral density. Curcumin, a diketone metabolite derived from *Curcuma longa* L. [Zingiberaceae], exhibits multifaceted effects, including improvement of osteoblast function and regulation of bone metabolism (Chen S. et al., 2021; Tagde et al., 2021; Yang et al., 2023; Dahiya et al., 2024). Fang et al. demonstrated that inhibition of hyperglycemia-activated NF-κB signaling contributes to the protective effect of curcumin on the impaired osteogenesis–angiogenesis coupling of bone marrow mesenchymal stem cells (BMSCs) under high-glucose conditions (Fan et al., 2022). Specifically, curcumin restores the pro-angiogenic capacity of BMSCs *in vitro* by upregulating VEGF expression, thereby promoting proliferation, migration, and tube formation of human umbilical vein endothelial cells (HUVECs); *in vivo*, it enhances type H vessel abundance and prevents diabetes-induced bone loss. However, curcumin is a pan-assay interfering substance (Magalhães et al., 2021), and its effects should be considered through integrated analysis of *in vitro* and *in vivo* studies, and even clinical data. Its poor solubility and low oral bioavailability also limit efficacy, suggesting future work should explore nanoparticle or alternative delivery strategies.

*Panax quinquefolium* saponin (PQS), extracted from the stems and leaves of *Panax quinquefolius* L. [Araliaceae], has been shown to improve myocardial ischemic injury and promote angiogenesis (Guo et al., 2018; Pan et al., 2024). In a hindlimb unloading mouse model, which is widely used to simulate disuse osteoporosis, researchers observed that PQS administration elevated serum levels of VEGF and Noggin, enhanced type H vessel formation, and ultimately increased bone mass (Liang et al., 2021). Further *in vitro* studies demonstrated that PQS treatment of human microvascular endothelial cells (HMECs) significantly enhanced VEGF and Noggin secretion, thereby promoting osteogenesis. Conversely, HMECs with VEGF and Noggin knockdown exhibited attenuated pro-osteogenic effects. These findings collectively indicate that VEGF and Noggin serve as critical mediators in PQS-induced coupling of type H vessel angiogenesis and osteogenesis. These findings have significant implications for treating osteoporosis caused by reduced mechanical stimulation in bedridden patients and astronauts; however, the study was limited to male hindlimb-unloaded mice, and future work could include female mice or estrogen-deficient OVX models to explore different types of osteoporosis.

Ginsenoside Rg1 (Rg1), a protopanaxatriol-type steroidal saponin and one of the most pharmacologically active components of PQS, exhibits pronounced anti-osteoporotic properties (Wu et al., 2021; Jiang et al., 2024). Chen et al. found that Rg1 exerts anti-osteoporotic effects through multiple mechanisms (Chen et al., 2022). *In vitro*, Rg1 counteracted high-glucose–induced inhibition, promoted HUVEC proliferation, and activated the Notch signaling pathway, stimulated endothelial cells to secrete the osteogenic factor Noggin, thereby regulating bone formation. It also facilitated VEGF secretion from osteoprogenitor cells, promoting the coupling of osteogenesis and angiogenesis. *In vivo*, Rg1 intervention in a diabetic osteoporosis rat model confirmed its ability to enhance VEGF expression, activate the Noggin/Notch pathway, increase type H vessel formation and bone formation, ultimately improving bone microarchitecture and increasing bone mass. Their study is the first to demonstrate that Rg1 enhances angiogenesis–osteogenesis coupling in diabetic osteoporosis, highlighting its potential as a therapeutic agent and broadening the application of bioactive metabolites for different types of osteoporosis.

Tetramethylpyrazine (TMP), a pyrazine alkaloid derived from the traditional Chinese botanical drug Conioselinum anthriscoides ‘Chuanxiong’ [Apiaceae], has demonstrated multiple pharmacological activities, including bone repair promotion and amelioration of steroid-induced osteonecrosis (Jiang et al., 2015; Ai and Zheng, 2025). Gao et al. investigated the role of TMP in eliminating senescent mesenchymal stem/progenitor cells in the bone marrow (Gao et al., 2018). They found that senescent LepR^+^ mesenchymal stem/progenitor cells (MSPCs) were markedly accumulated in the bone marrow of aged mice. TMP significantly suppressed the senescence phenotype of these cells by modulating the Ezh2-H3K27me3 axis. Importantly, local delivery of TMP improved the bone marrow microenvironment by enhancing metabolic activity and anti-inflammatory responses, inducing type H vessel formation, and maintaining the hematopoietic stem cell niche, thereby preserving bone homeostasis in aged mice. With aging, many mesenchymal stem cells become dysfunctional and enter a state of senescence, leading to disruption of the bone marrow microenvironment, including impaired hematopoietic stem cell maintenance, release of inflammatory cytokines, and compromised angiogenesis and osteogenesis, which collectively contribute to age-related osteoporosis. Local delivery of TMP activates AMPK/mTOR signaling phosphorylation in type H endothelial cells and upregulates the HIF-1α/VEGF signaling axis, thereby increasing the abundance of type H vessels, which plays a crucial role in mitigating bone loss (Gao et al., 2018). However, this study employed intramedullary TMP administration, and future work could investigate bone-targeted delivery using tissue engineering strategies, which may offer a more clinically feasible approach for age-related bone disorders.

Daidzein is a natural isoflavone metabolite derived from soybeans with significant anti-osteoporotic activity (Bellavia et al., 2021; Laddha and Kulkarni, 2023). Previous studies have shown that Caveolin-1 (Cav-1) is a functional regulator of osteoclast differentiation, and silencing Cav-1 can inhibit osteoclast formation and prevent OVX-induced osteoporosis (Lee et al., 2015; Zou et al., 2021). Notably, Daidzein has been identified as a specific inhibitor of Cav-1 (Xie et al., 2019; Liu et al., 2020). The study by Jia et al. revealed the mechanism of Daidzein: *in vitro* experiments showed that Daidzein significantly enhanced the migratory and proliferative capacities of bone marrow endothelial cells (BMECs) through Cav-1 inhibition-mediated activation of the EGFR/PI3K/AKT signaling pathway; in OVX mouse models, Daidzein treatment effectively promoted type H vessel formation and significantly increased bone mass (Jia et al., 2023). These findings not only elucidate the mechanism by which Daidzein promotes angiogenesis and osteogenesis through Cav-1 inhibition, but also provide important experimental evidence for developing natural product-based therapeutic strategies for osteoporosis. However, these molecular mechanisms still need further investigation *in vivo*, and future work should explore them to better understand Daidzein’s anti-osteoporotic effects.

**FIGURE 1:**
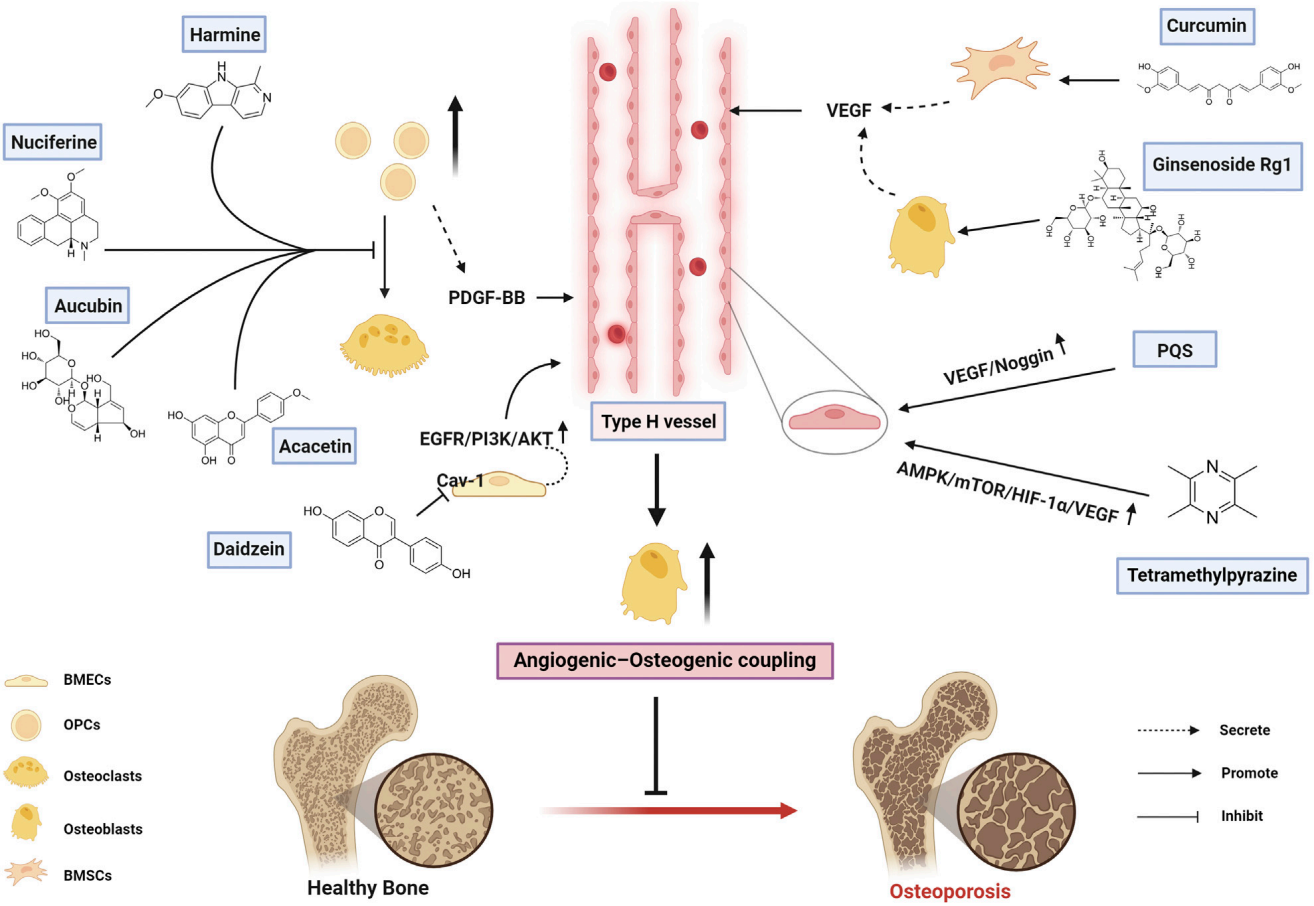
Therapeutic mechanisms of bioactive metabolites targeting type H vessels in osteoporosis treatment (Created with BioRender.com).

The mechanisms by which bioactive metabolites regulate type H vessels for osteoporosis treatment are systematically summarized in Figure 1 and Table 2.

**TABLE 2:** Therapeutic mechanisms of bioactive metabolites targeting type H vessels in osteoporosis treatment.

Metabolites	Sources	Dose and duration	Model and administration	Target cells	Molecular mechanism	Therapeutic effects	References
Harmine	*Peganum harmala* L. [Nitrariaceae]	10 mg/kg/day; 2 months	OVX mice (intragastric gavage)	OPCs	Increases PDGF-BB expression	Inhibits osteoclast maturation, enhances type H vessel formation, and promotes bone formation	Huang et al. (2018)
Nuciferine	*Nelumbo nucifera* Gaertn. [Nelumbonaceae]	40 mg/kg/day; 2 months	OVX mice (intragastric gavage)	OPCs	Inhibits NF-κB signaling, increases PDGF-BB production	Reduces osteoclast differentiation, stimulates type H vessel angiogenesis, and enhances osteogenesis	Song et al. (2020)
Aucubin	*Aucuba japonica* Thunb. [Garryaceae]; *Eucommia ulmoides* Oliv. [Eucommiaceae]	5 mg/kg every other day; 1 month	OVX mice (intraperitoneal injection)	OPCs	Inhibits NF-κB pathway, elevates PDGF-BB levels	Suppresses osteoclastogenesis, increases type H vessel density, and improves bone mass	Li et al. (2022)
Acacetin	*Vachellia farnesiana* (L.) Wight & Arn. [Fabaceae]	20 mg/kg/day; 8 weeks	OVX mice (intragastric gavage)	OPCs	Inhibits NF-κB and Akt/GSK3β pathways, enhances PDGF-BB release	Promotes type H vessel formation, stimulates bone formation	Lin et al. (2022)
Curcumin	*Curcuma longa* L. [Zingiberaceae]	100 mg/kg/day; 8 weeks	Diabetic mice (intragastric gavage)	BMSCs	Stimulates VEGF secretion	Reverses BMSCs’ dysfunction, promotes osteoblast differentiation, and enhances type H vessel formation	Fan et al. (2022)
Panax quinquefolium saponin	*Panax quinquefolius* L. [Araliaceae]	15, 45, 135 mg/kg/day; 4 weeks	HU mice (intragastric gavage)	Type H ECs	Activates Noggin/VEGF signaling	Increases type H vessel number, enhances bone formation	Liang et al. (2021)
Ginsenoside Rg1	*Panax ginseng* C.A.Mey. [Araliaceae]; *Panax quinquefolius* L. [Araliaceae]	10 mg/kg/day; 12 weeks	Diabetic rats (intragastric gavage)	Type H ECs and osteoblasts	Activates Notch/Noggin/VEGF pathway	Stimulates type H vessel angiogenesis, improves bone mass	Chen et al. (2022)
Tetramethylpyrazine	*Conioselinum anthriscoides* ‘Chuanxiong’ [Apiaceae]	10 μg/kg; 3 times/week; 8 weeks	Aged mice (intramedullary injection)	LepR^+^ MSPCs and type H ECs	Upregulates AMPK/mTOR and HIF-1α/VEGF axis	Enhances type H vessel generation, maintains the marrow microenvironment	Gao et al. (2018)
Daidzein	*Glycine max* (L.) Merr. [Fabaceae]	25 mg/kg; 5 days/week; 8 weeks	OVX mice (intragastric gavage)	BMECs	Inhibits Cav-1 protein, activates EGFR/PI3K/AKT signaling	Stimulates type H vessel angiogenesis, increases bone mass	Jia et al. (2023)

### 3.2 Osteoarthritis

Osteoarthritis (OA) is a chronic degenerative disease involving all joint structures, including articular cartilage, subchondral bone, synovium, ligaments, and joint capsule, characterized by pathological changes such as cartilage wear and degeneration, subchondral bone remodeling, synovial inflammation, and osteophyte formation. Clinically, OA primarily affects weight-bearing joints like the hips and knees, presenting with chronic pain and joint instability. The disease shows marked gender and age disparities, being more prevalent in women and elderly populations, with approximately 60% of people over 50 years affected, increasing to 80% in those over 75 years, where disability rates reach 53% (Zhang Z. et al., 2020). Major risk factors include aging, obesity, joint injury, and trauma, among which aging and obesity are considered the most significant (Zhang Z. et al., 2020). Recent studies have identified pathological type H vessel formation-induced subchondral bone ossification as a significant pathogenic factor in OA (Liu Y. et al., 2023). Current pharmacotherapies for OA primarily include analgesics, articular cartilage protectants (Richard et al., 2023). However, long-term NSAID use may induce gastrointestinal complications and cardiovascular risks. Emerging research suggests that bioactive metabolites targeting abnormal type H vessel proliferation in subchondral bone can pathologically ameliorate osteophyte formation, representing a novel therapeutic strategy for OA management.

Several bioactive metabolites have shown promising therapeutic potential for OA, among which Soybean Isoflavones (SI) represent an important candidate. As a class of flavonoid metabolites derived from soybeans, SI exhibits multiple pharmacological activities against bone disorders (Li L. L. et al., 2023; Valizadeh et al., 2025). Zou et al. conducted an in-depth investigation into SI’s ability to reduce abnormal subchondral bone hyperplasia (Zou et al., 2024). They administered SI by intragastric gavage in an OA rat model established *via* destabilization of the medial meniscus (DMM) surgery and found that SI reduced joint damage and OARSI scores, indicating that SI alleviated cartilage degeneration and delayed OA progression. Further experiments revealed that SI downregulated the expression of ALP, OCN, and BMP in the subchondral bone region. Importantly, SI upregulated the expression of Tuberous Sclerosis Complex 1 (TSC1) and inhibited the mTORC1 signaling pathway, thereby suppressing excessive osteoblast activation and VEGF release, leading to the inhibition of type H vessel formation and attenuation of aberrant subchondral bone remodeling. These findings suggest a novel therapeutic strategy for OA by targeting subchondral bone remodeling and suppressing the angiogenesis–osteogenesis coupling process, thereby delaying disease progression and improving the structural and functional integrity of the osteoarticular system. Further work is needed to evaluate its pharmacokinetics, optimal dosing, and long-term efficacy *in vivo* to support its potential therapeutic use in OA.

Halofuginone (HF), a quinazolinone alkaloid derived from the traditional Chinese medicinal plant Dichroa febrifuga with a history of use in Chinese herbal medicine that spans over 2,000 years, has transitioned from its original antimalarial application to become a promising therapeutic agent for bone disorders (Zhu et al., 2024; Tang et al., 2025). Cui et al. investigated the effects of HF in OA mouse models induced by anterior cruciate ligament transection (ACLT) *via* intraperitoneal injection (Cui et al., 2016). They found that HF significantly alleviated articular cartilage degeneration and subchondral bone deterioration. HF reduced the expression of collagen type X (ColX) and matrix metalloproteinase-13 (MMP-13), while increasing the expression of collagen type II and aggrecan. It also decreased subchondral bone volume and increased the thickness of the subchondral bone plate, thereby suppressing aberrant subchondral ossification. Mechanistically, HF attenuated osteoclast maturation by inhibiting RANKL expression and restraining the excessive formation of type H vessels in the subchondral bone by downregulating Smad2/3-dependent TGF-β signaling, ultimately delaying the progression of osteoarthritis. Collectively, HF shows promise in targeting subchondral bone remodeling and angiogenesis in OA, and future studies should further explore its clinical efficacy.

Isoliquiritigenin (ISL), a chalcone-type flavonoid metabolite derived from licorice, exhibits dual pharmacological activities by simultaneously inhibiting osteoclastogenesis and angiogenesis (Wang et al., 2019; Jeong et al., 2020; Joyce et al., 2022). Ji et al. treated ACLT mouse models with intraperitoneal ISL injection and found that ISL significantly increased lubricin expression while reducing the levels of ColX and MMP-13, effectively inhibiting abnormal subchondral bone remodeling (Ji et al., 2018). ISL alleviated OA progression and protected articular cartilage through two mechanisms. First, it suppressed abnormal subchondral bone remodeling in early OA by reducing osteoclastogenesis induced by the RANKL-RANK-TRAF6 signaling pathway and preventing abnormal bone formation through the inhibition of TGF-β release. Second, ISL directly inhibited MMP-2 and VEGFR-2 expression, thereby preventing the formation of aberrant type H vessels in the subchondral bone. These findings suggest that ISL is a promising natural compound that targets early OA subchondral bone, demonstrates significant efficacy in ACLT mouse models, preserves subchondral bone integrity, and warrants future clinical investigation as a potential preventive therapy for OA.

Loganin is an active iridoid glycoside metabolite from Cornus officinalis Siebold & Zucc. [Cornaceae], a traditional Chinese medicinal botanical drug with both dietary and therapeutic value, demonstrates multi-faceted potential in treating skeletal diseases (Hu J. et al., 2020; Hu et al., 2025). Building upon the traditional Chinese medical applications of Corni Fructus-containing formulations in OA treatment, Hu et al. demonstrated through intra-articular administration in OA mouse models that Loganin significantly enhances hyaline cartilage thickness while suppressing calcified cartilage formation (Hu J. et al., 2020). The mechanistic investigation revealed that Loganin mediates its chondroprotective effects by coordinately upregulating type II collagen (Col2) expression and downregulating matrix metalloproteinase-13 (MMP13) and type X collagen (Col10) levels in articular cartilage tissue. Importantly, Loganin directly suppresses the formation of type H vessels in the subchondral bone and also alleviates osteophyte development, cartilage matrix degradation, and chondrocyte pyroptosis through inhibition of the NF-κB signaling pathway. These findings highlight Loganin as a promising therapeutic option for osteoarthritis. However, this study primarily elucidated the effects of Loganin on osteoarthritic chondrocytes, and the direct molecular mechanism by which Loganin regulates type H vessel formation in the subchondral bone remains to be clarified, representing a valuable direction for future research.

Diterbutyl phthalate (DP), an ester metabolite derived from Panax notoginseng (Sanqi). In osteoarthritis pathogenesis, abnormal mechanical loading triggers excessive osteoclastogenesis and bone resorption in subchondral bone, leading to cyst formation, sclerosis, and ultimately articular cartilage degeneration. Fang et al. demonstrated through both OA mouse models and *in vitro* experiments that DP treatment inhibits pathological osteoclast fusion in subchondral bone by suppressing the ERK/c-fos/NFATc1 pathway and downregulating key osteoclast fusion regulators (DC-STAMP and Atp6v0d2), thereby ameliorating cartilage degeneration (Fang et al., 2022). Furthermore, DP attenuates abnormal type H vessel formation in subchondral bone through MMP-2 inhibition, resulting in pain relief. These findings collectively establish DP as a promising therapeutic agent targeting both aberrant osteoclast activity and type H vessel generation in OA progression. Through further optimization and formulation improvements, DP may serve as a basis for developing innovative therapies for osteoarthritis, and as a potential preventive agent, it demonstrates broad prospects for clinical application in mitigating OA onset and progression.

Resveratrol, a naturally occurring non-flavonoid polyphenol found in grapes, blueberries, and other dietary sources, exhibits potent antioxidant and anti-inflammatory properties (Meng et al., 2020). In an ACLT-induced OA mouse model, Xiong et al. demonstrated that intraperitoneal Resveratrol administration exerts dual protective effects by suppressing type H vessel formation to preserve articular cartilage structure and delay cartilage degeneration, while simultaneously inhibiting osteoclast differentiation and activity to reduce pathological osteoclast accumulation and bone resorption in subchondral bone (Xiong et al., 2021). Mechanistically, Resveratrol modulates the OPG/RANKL/RANK pathway to suppress osteoclastogenesis and reduce bone resorption while downregulating VEGF-A and Angiopoietin-1 expression to inhibit aberrant type H vessel generation, thereby disrupting the pathological angiogenesis-osteogenesis coupling and ultimately slowing OA progression. By simultaneously targeting multiple components of the vascular-bone-cartilage network, Resveratrol not only provides novel insights into OA pathogenesis but also represents a promising foundation for developing natural product-based therapeutic strategies against OA. However, as Resveratrol is a pan-assay interfering substance (Magalhães et al., 2021), and the study was conducted exclusively *in vivo* with limited quantitative analysis of staining and no complementary *in vitro* validation, further investigations using molecular and cellular approaches would be valuable to clarify its direct targets and confirm its therapeutic potential.

**FIGURE 2:**
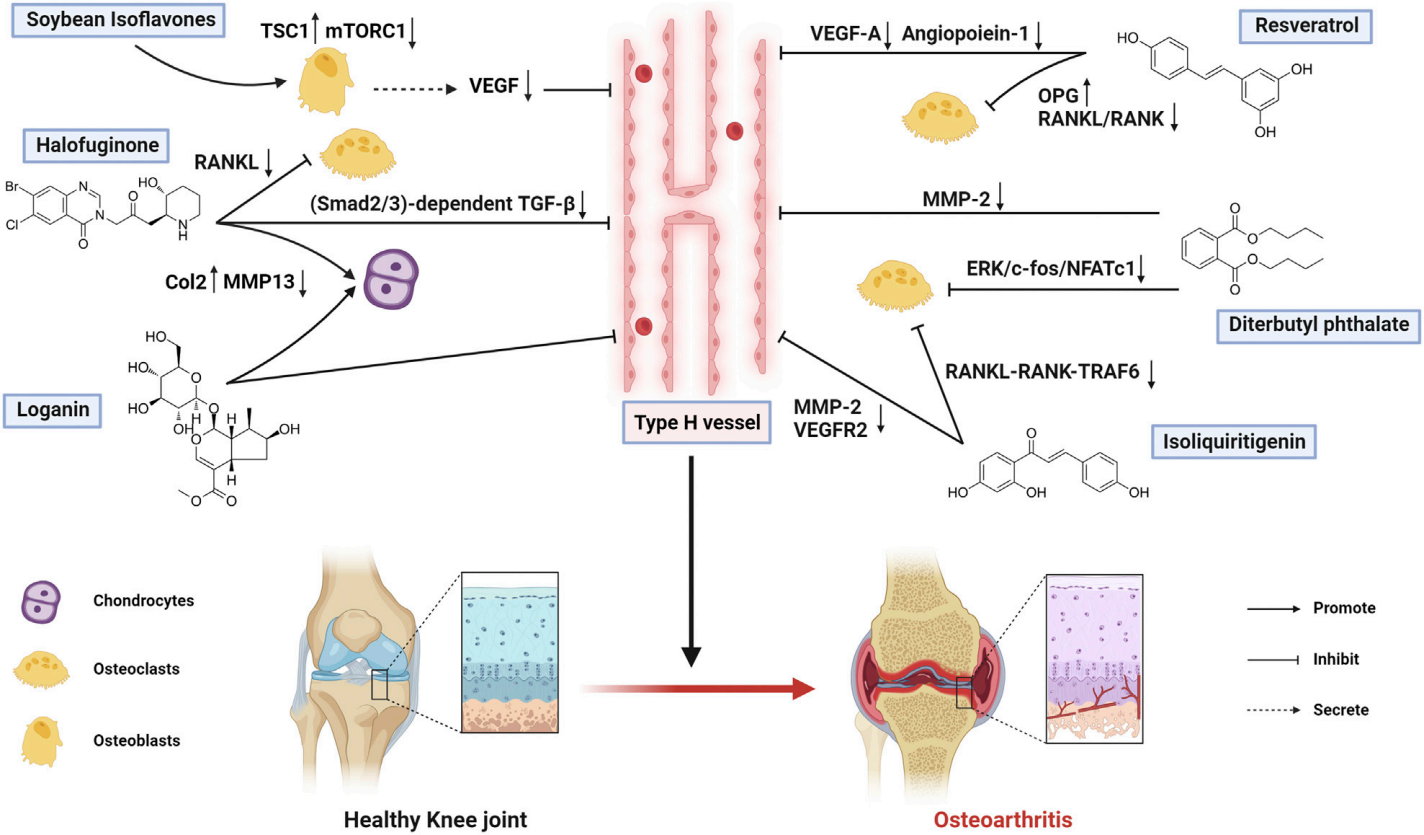
Therapeutic mechanisms of bioactive metabolites targeting type H vessels in osteoarthritis treatment (Created with BioRender.com).

The mechanisms by which bioactive metabolites regulate type H vessels for osteoarthritis treatment are systematically summarized in Figure 2 and Table 3.

**TABLE 3:** Therapeutic mechanisms of bioactive metabolites targeting type H vessels in osteoarthritis treatment.

Metabolites	Sources	Dose and duration	Model and administration	Target cells	Molecular mechanism	Therapeutic effects	References
Soybean Isoflavones	*Glycine max* (L.) Merr. [Fabaceae]	50 mg/kg/day; 2 weeks	DMM-induced OA rats (intragastric gavage)	Osteoblasts	Upregulates TSC1, downregulates mTORC1, inhibits VEGF	Suppresses type H vessel formation, attenuates subchondral bone remodeling, and reduces OA progression	Zou et al. (2024)
Halofuginone	*Hydrangea febrifuga* (Lour.) Y.De Smet and Granados [Hydrangeaceae]	1 mg/kg every other day; 1 month	ACLT-induced OA mice (intraperitoneal injection)	Osteoclasts, Chondrocytes, and type H ECs	Inhibits Smad2/3-dependent TGF-β signaling	Reduces type H vessels, prevents pathological subchondral ossification, and alleviates OA	Cui et al. (2016)
Isoliquiritigenin	*Glycyrrhiza glabra* L. [Fabaceae]	40 mg/kg every other day; 60 days	ACLT-induced OA mice (intraperitoneal injection)	Osteoclasts and type H ECs	Inhibits RANKL-RANK-TRAF6 and TGF-β, downregulates MMP-2 and VEGFR2	Blocks osteoclast maturation, decreases type H vessels in subchondral bone, and mitigates OA	Ji et al. (2018)
Loganin	*Cornus officinalis* Siebold and Zucc. [Cornaceae]	30, 100 μg/mL, twice a week; 8, 12 weeks	DMM-induced OA mice (intra-articular injection)	Chondrocytes and type H ECs	Upregulates Col2, downregulates MMP13 and Col10, inhibits NF-κB	Reduces cartilage catabolism and chondrocyte pyroptosis, inhibits type H vessels, and ameliorates OA	Hu et al. (2020a)
Diterbutyl phthalate	*Panax notoginseng* (Burkill) F.H.Chen [Araliaceae]	5 mg/kg/day; 8 weeks	ACLT-induced OA mice (intraperitoneal injection)	Osteoclasts and type H ECs	Suppresses RANKL-induced ERK/c-fos/NFATc1, inhibits MMP-2	Prevents osteoclast fusion, reduces aberrant osteoclasts and type H vessels, alleviates OA	Fang et al. (2022)
Resveratrol	*Reynoutria japonica* Houtt. [Polygonaceae]; *Vitis labrusca* L. [Vitaceae]	50 mg/kg; 60 days	ACLT-induced OA mice (intraperitoneal injection)	Osteoclasts and type H ECs	Regulates OPG/RANKL/RANK, downregulates VEGF-A and Angiopoietin-1	Inhibits osteoclast differentiation/bone resorption, suppresses type H vessels, delays cartilage degeneration	Xiong et al. (2021)

### 3.3 Bone defects and fracture

Bone defects, characterized by partial or complete loss of bone tissue, arise from diverse etiologies, including trauma with soft tissue/periosteal damage and post-tumor resection, often resulting in severe bone loss (Nauth et al., 2018). Bone defects frequently correlate with nonunion and may progress to infectious osteomyelitis when complicated by infection (Budiatin et al., 2021; Liu B. et al., 2024). Current treatments (bone grafting, Ilizarov technique, Masquelet technique) face challenges such as limited graft availability, malunion, and postoperative infections (Aktuglu et al., 2019; Baldwin et al., 2019; Alford et al., 2021). Fractures occur when mechanical loads exceed the bone’s load-bearing threshold, disrupting skeletal continuity, which may result from either acute high-intensity trauma or chronic cumulative/repetitive microdamage, with additional contributions from pathological conditions (e.g., osteoporosis) and age-related degeneration. The primary objectives of fracture treatment are anatomical reduction and functional restoration; however, postoperative fracture nonunion occurs in 5%–10% of cases, with incidence rates escalating to 30% in patients with concomitant open injuries, immune disorders, or osteoporosis (Hak et al., 2014; Moghaddam et al., 2016). Bone defect repair and fracture healing share remarkable similarities, both relying on the mechanism of “angiogenesis-osteogenesis coupling.” In this process, the formation of new blood vessels not only supplies nutrients and oxygen to bone tissue but also secretes growth factors that support bone formation and remodeling, thereby restoring the structural integrity and mechanical function of bone (Xu et al., 2024). Moreover, the molecular mechanisms underlying bone defect repair and fracture healing are closely associated with the activation of signaling pathways such as Wnt/β-catenin and BMP/TGF-β (Majidinia et al., 2018). Currently, pharmacological treatments for bone defects and fractures primarily focus on calcium supplementation, regulation of bone metabolism, infection control, and pain relief; however, there is still a lack of effective pro-angiogenic agents (Vannucci and Brandi, 2016; Fischer et al., 2018; Gupta et al., 2020). Emerging evidence indicates that certain bioactive metabolites can enhance bone regeneration by modulating type H vessel formation.

Astragaloside IV (AS-IV), a cycloartane-type triterpene glycoside derived from the traditional Chinese medicine Astragalus membranaceus, exhibits multifaceted pharmacological properties, including pro-angiogenic activity and antioxidative stress (Liang et al., 2023; Ou et al., 2023). In a rat model of distraction osteogenesis (DO), intragastric gavage of AS-IV demonstrated remarkable pro-angiogenic effects during bone regeneration, significantly increasing type H vessel density at defect sites while improving BMD and bone volume/tissue volume (BV/TV). Mechanistic studies revealed that AS-IV activates the AKT/GSK-3β/β-catenin pathway to suppress multinucleated osteoclast differentiation while enhancing PDGF-BB secretion from OPCs, thereby stimulating type H vessel formation. Furthermore, AS-IV was shown to promote BMSCs’ viability, osteogenic differentiation, and expression of pro-angiogenic genes, collectively demonstrating its ability to reinforce angiogenesis-osteogenesis coupling for bone defect repair (Wang et al., 2021). These findings highlight AS-IV’s potential in promoting angiogenesis-osteogenesis coupling, while future studies should further explore its pharmacokinetic characteristics, safety profile, and optimal administration in large animal models.

Ophiopogonin D, a naturally occurring C29 steroidal glycoside derived from Ophiopogon japonicus (Thunb.) Ker Gawl. [Asparagaceae], exhibits significant pharmacological potential in bone and cardiovascular protection (Chen et al., 2024). Yang et al. demonstrated using endothelial-specific KLF3 knockout mice subjected to surgical trabecular bone ablation that genetic KLF3 deletion markedly enhances type H vessel formation in bone regeneration areas, accelerating osteogenesis (Yang et al., 2020). Subsequent investigations in wild-type mice with trabecular bone defects revealed that intraperitoneal Ophiopogonin D administration promotes type H vessel generation and bone repair by inhibiting KLF3, thereby relieving its transcriptional repression of JUNB and downstream VEGF-A expression. *In vitro* cell experiments showed that treatment with Ophiopogonin D enhanced the migration and tube formation abilities of HMECs, but had no effect on the migration of BMSCs, osteogenic differentiation, or osteoclast differentiation. This indicates that Ophiopogonin D primarily promotes the formation of type H vessels by targeting vascular endothelial cells, thereby indirectly facilitating bone regeneration. Notably, this study employed endothelial-specific KLF3 knockout mice to dissect the role of type H vessels in bone regeneration, providing robust genetic evidence that strengthens the mechanistic link between Ophiopogonin D, KLF3 inhibition, and enhanced angiogenesis-mediated bone repair.

Total Flavonoids of Rhizoma Drynariae (TFRD), a flavonoid complex derived from the traditional Chinese medicine Rhizoma Drynariae, exhibits potent osteogenic and anti-inflammatory properties that contribute to its therapeutic effects on bone defects. Shen et al. established rat distraction DO and fracture models, providing the first evidence that DO exhibits significantly higher type H vessel abundance than fracture (Shen et al., 2020; Shen et al., 2022). The study demonstrated that TFRD potently stimulates type H vessel formation *via* the PDGF-BB/PDGFR-β axis (rather than the HIF-1α/VEGF pathway), activating downstream p-AKT/p-ERK1/2 to enhance both the angiogenic capacity of EPCs and osteogenic differentiation of BMSCs. Furthermore, TFRD upregulated the PDGF-BB/VEGF/RUNX2/OSX signaling cascade to effectively promote “angiogenesis-osteogenesis coupling,” an effect reversible by PDGF-BB neutralizing antibodies. Their study provides a theoretical foundation for accelerating callus formation and mineralization during DO, thereby shortening the bone consolidation period. Nowadays, TFRD has been developed into a postmarketing traditional Chinese medicine called Qianggu Capsule, with the drug approval number Z20030007, and its established safety and accessibility support its clinical application in bone disease therapy. Moreover, these findings offer a valuable paradigm for the Ilizarov technique in bone disease treatment, with the therapeutic effect likely attributed to the promotion of type H vessel–mediated angiogenesis.

Type 1 diabetes mellitus (T1DM)-induced chronic hyperglycemia leads to diabetic osteopathy, characterized by disrupted bone metabolism and vascular abnormalities, particularly impaired type H vessel formation that severely compromises bone regenerative capacity (Napoli et al., 2017; Hu et al., 2021). Icariin (ICA) is a natural flavonoid glycoside extracted from Epimedium, with a variety of pharmacological activities, including promoting osteogenesis, inhibiting osteoclasts, and protecting cartilage (Bai et al., 2023; Zeng et al., 2023; Mohammadzadeh et al., 2024; Si et al., 2024). Zheng et al.demonstrated in a streptozotocin-induced T1DM rat model with surgically created bone defects that oral ICA administration effectively restored type H vessel formation at defect sites, subsequently enhancing bone regeneration (Zheng et al., 2024b). Mechanistically, ICA upregulates both osteogenic markers (ALP, OCN) and angiogenic factors (VEGF-A, CD31) in BMSCs, thereby promoting the coupling between type H vessel formation and osteogenesis. These findings position ICA as a promising therapeutic candidate for diabetic bone repair through its ability to reactivate the impaired angiogenesis-osteogenesis coupling process under diabetic conditions.

Ginsenoside Compound K (CK), an intestinal bacterial metabolite derived from ginsenosides Rb1, Rb2, and Rc belonging to the protopanaxadiol-type saponins, demonstrates significant potential in bone repair (Morshed et al., 2024). Previous studies have established its ability to activate Wnt/β-catenin signaling, the canonical pathway governing osteogenesis and osteoblast differentiation (Zhou et al., 2018; Shi et al., 2020). Ding et al. demonstrated through local injection of CK at fracture sites in rat models of open femoral fracture, combined with *in vitro* experiments, that CK exhibits a dual therapeutic mechanism in fracture repair (Ding et al., 2022). CK not only activates the Wnt/β-catenin signaling pathway to promote osteogenic differentiation of BMSCs, but also enhances the angiogenic capacity of endothelial cells, significantly increasing type H vessel formation at the fracture site. This coordinated action effectively improves bone healing quality in open femoral fractures, as evidenced by enhanced callus formation and significantly improved biomechanical strength, thereby providing crucial theoretical support for the clinical application of CK.

Osteoporotic fractures represent a prevalent fracture subtype, with growing research attention on the potential of bioactive metabolites to enhance type H vessel formation for their treatment. Osthole, a coumarin derivative isolated from *Cnidium monnieri* (L.) Cusson [Apiaceae], has demonstrated multiple pharmacological properties, including anti-osteoporotic and anti-inflammatory effects (Wang P. et al., 2017; Zheng et al., 2019). Zheng et al. investigated Osthole‘s therapeutic potential using an OVX rat tibial fracture model combined with *in vitro* studies, revealing that Osthole could not directly promote angiogenesis in HUVECs, but could facilitate BMSC-mediated angiogenesis (Zheng et al., 2024a). Mechanistically, Osthole activates the Wnt/β-catenin pathway in BMSCs to enhance their osteogenic differentiation, while upregulating VEGF-A expression levels, thereby inducing type H vessel formation and reinforcing the “angiogenesis-osteogenesis coupling”. These effects collectively accelerate osteoporotic fracture healing, suggesting Osthole may serve as a potential therapeutic agent for osteoporotic fractures. Future studies should include toxicological evaluation and human clinical trials to fully confirm Osthole’s safety and therapeutic potential.

**FIGURE 3:**
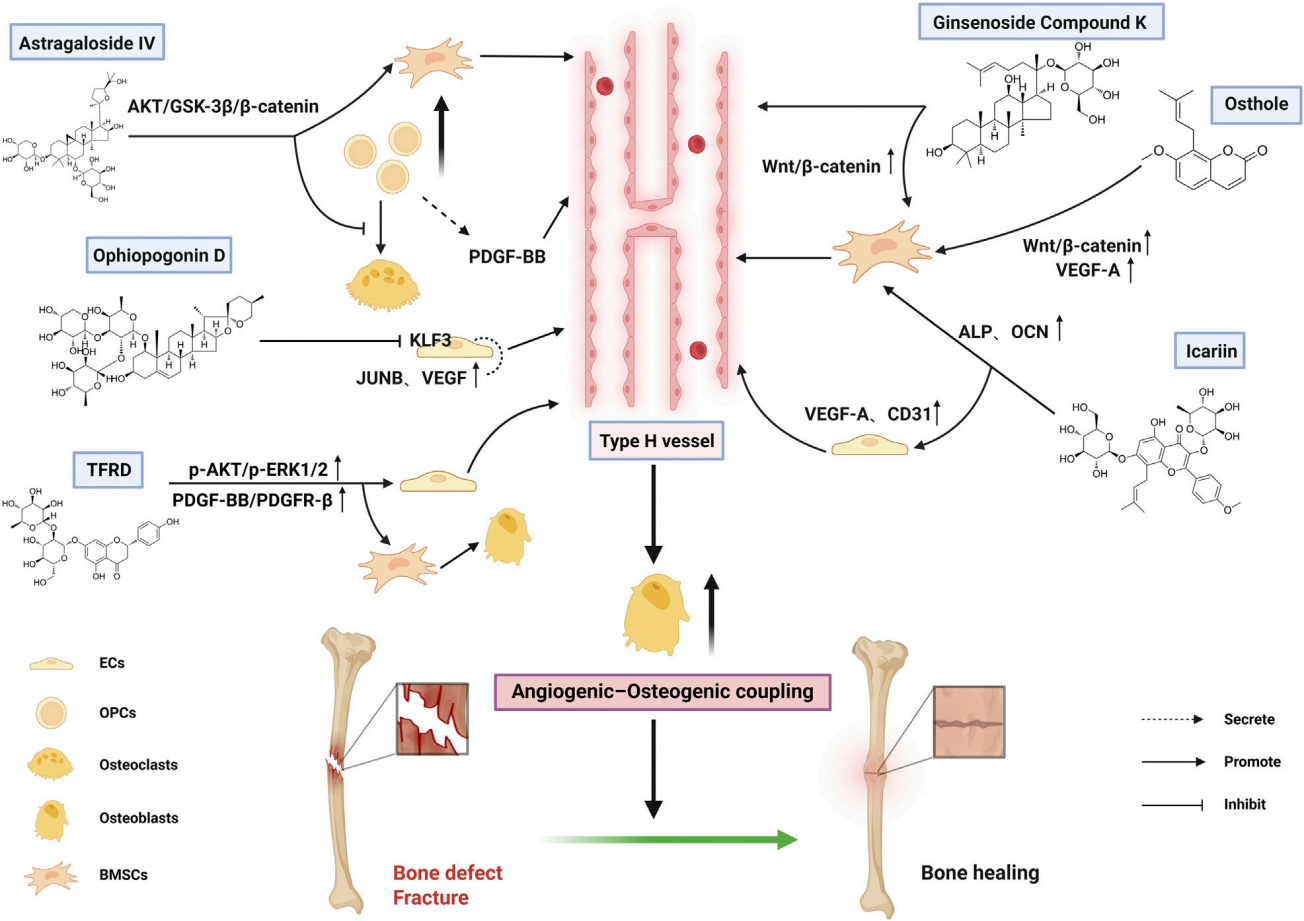
Therapeutic mechanisms of bioactive metabolites targeting type H vessels in bone defects and fracture treatment (Created with BioRender.com).

The mechanisms by which bioactive metabolites regulate type H vessels for Bone defects and Fracture treatment are systematically summarized in Figure 3 and Table 4.

**TABLE 4:** Therapeutic mechanisms of bioactive metabolites targeting type H vessels in bone defects and fracture treatment.

Metabolites	Sources	Dose and duration	Model and administration	Target cells	Molecular mechanism	Therapeutic effects	References
Astragaloside IV	*Astragalus mongholicus* Bunge [Fabaceae]	20 mg/kg/day; 4 weeks	DO rat model (intragastric gavage)	BMSCs and Osteoclasts	Activates AKT/GSK-3β/β-catenin pathway and enhances PDGF-BB expression	Increases PDGF-BB production from OPCs, promotes BMSCs’ osteogenic differentiation and angiogenic gene expression, elevates type H vessel density, and accelerates bone regeneration	Wang et al. (2021)
Ophiopogonin D	*Ophiopogon japonicus* (Thunb.) Ker Gawl. [Asparagaceae]	20 mg/kg/day; 7 days	Femoral trabecular ablation mice (intraperitoneal injection)	ECs	Inhibits KLF3 to relieve its transcriptional repression on JUNB and downstream VEGF-A	Promotes type H vessel formation *in vivo* and enhances bone regeneration	Yang et al. (2020)
Total Flavonoids of Rhizoma Drynariae	*Drynaria roosii Nakaike* [Polypodiaceae]	75 mg/kg/day; 17, 45 days	DO/Fracture rat model (intragastric gavage)	ECs and BMSCs	Activates PDGF-BB/PDGFR-β signaling and downstream AKT/ERK1/2	Stimulates type H vessel generation, improves bone repair, and accelerates regeneration	Shen et al. (2020)
Icariin	*Epimedium sagittatum* (Siebold and Zucc.) Maxim. [Berberidaceae]	100 mg/kg/day; 4 weeks	Diabetic bone defect rats (intragastric gavage)	ECs and BMSCs	Upregulates expression of osteogenic markers (ALP, OCN) and angiogenic factors (VEGF-A, CD31)	Enhances type H vessel formation at defect sites and promotes bone regeneration	Zheng et al. (2024b)
Ginsenoside Compound K	*Panax ginseng* C.A.Mey. [Araliaceae]; *Panax quinquefolius* L. [Araliaceae]	500 μM, 100 μL, every other day; 4 weeks	Open femoral fracture rats (local injection)	Type H ECs and BMSCs	Activates Wnt/β-catenin signaling pathway	Increases callus formation, elevates type H vessel density, and improves fracture healing	Ding et al. (2022)
Osthole	*Cnidium monnieri* (L.) Cusson [Apiaceae]	10 mg/kg/day; 6 weeks	OVX tibial fracture rats (intragastric gavage)	BMSCs	Activates Wnt/β-catenin signaling pathway, upregulates VEGF-A	Promotes osteogenic differentiation of BMSCs and increases type H vessel abundance, accelerating fracture healing	Zheng et al. (2024a)

### 3.4 Osteonecrosis of the femoral head

Osteonecrosis of the femoral head (ONFH) is a prevalent and challenging condition in orthopedics. Its global incidence continues to rise each year, with an estimated 8.12 million cases of non-traumatic ONFH in China alone (Zhao et al., 2020). Etiologically, ONFH is categorized as traumatic or non-traumatic, while the age of onset distinguishes adult ONFH from pediatric forms such as Legg-Calvé-Perthes disease (LCPD). Traumatic ONFH typically follows femoral neck fractures or hip dislocations and has clearly identified causes. In contrast, the mechanisms underlying non-traumatic ONFH remain incompletely understood, though prolonged glucocorticoid therapy and heavy alcohol intake are established major risk factors. LCPD is an idiopathic avascular necrosis of the femoral head epiphysis with a complex, multifactorial etiology (Zheng et al., 2025). Despite these differences, all ONFH subtypes share a common pathogenic pathway: disruption of the femoral head blood supply leads to ischemic necrosis of bone cells and marrow components, resulting in progressive structural deterioration. This vascular insufficiency compromises mechanical integrity, culminating in femoral head collapse. However, unlike adult ONFH, LCPD exhibits a pronounced self-limiting course, during which the femoral head can undergo spontaneous revascularization. Understanding the distinct regenerative capacities and underlying vascular mechanisms between adult ONFH and pediatric LCPD is crucial for developing targeted therapeutic strategies that can either restore blood supply or enhance reparative processes.

A recent single-cell transcriptome study revealed that type H vessels gradually decrease during the pathological progression of alcohol-induced ONFH (Shi et al., 2025). Animal studies have demonstrated that supraphysiological doses of steroid hormones can damage type H vessels and disrupt the angiogenesis-osteogenesis coupling, contributing to the development of steroid-induced ONFH (Ma et al., 2024; Zhang G. et al., 2024). In a lipopolysaccharide/methylprednisolone-induced ONFH rabbit model, Cao et al. showed that recombinant human PDGF-BB treatment restored femoral head blood supply by increasing type H vessel density (Cao et al., 2023). Similarly, a tissue engineering study found that injecting an miRNA-loaded hydrogel promotes bone regeneration in a rat model of ONFH by facilitating VAO-mediated type H vessel formation (Quan et al., 2025). Collectively, these findings suggest that promoting type H vessel formation may represent a promising therapeutic strategy for ONFH. However, no studies have yet investigated bioactive metabolites specifically targeting type H vessels in adult ONFH.

Currently, clinically effective pharmacological treatments to accelerate femoral head repair in LCPD are lacking. Notably, bioactive metabolites that promote type H vessel formation represent a promising therapeutic strategy. LCPD primarily affects children aged 4–8 years, with a natural disease course of approximately 2–4 years, coinciding with the period of high type H vessel abundance. In addition to the involvement of the femoral head epiphysis, the metaphyseal region also undergoes pathological changes in LCPD; however, these metaphyseal changes gradually regress and eventually resolve during disease progression (Li Z. et al., 2025). Considering that type H vessels are highly enriched in the metaphyseal regions of long bones, we speculate that they may play a role in this reparative process. Furthermore, endothelial dysfunction mediates impaired angiogenesis in LCPD, and treatment with the bioactive metabolite Biochanin A (BCA) can alleviate endothelial cell dysfunction induced by the inflammatory cytokine IL-6 (Li B. et al., 2021; Liu et al., 2024b; Liu et al., 2024c). BCA is an isoflavonoid metabolite that can be isolated from red clover, chickpeas, and soybeans and possesses multiple pharmacological activities, including antitumor and anti-inflammatory effects, as well as inhibition of osteoclast differentiation. (Liao et al., 2021; Sohel, 2024). Building on these findings, our team previously established a juvenile ischemic osteonecrosis (JIO) mouse model and evaluated the therapeutic effects of BCA (Huang Q. et al., 2025). This mouse model was originally developed by Prof. Kim’s team and effectively recapitulates the pathological process of LCPD (Kamiya et al., 2015). Our results showed that BCA markedly prevented epiphyseal collapse and promoted bone regeneration. Mechanistically, BCA enhances type H vessel formation in bone by indirectly promoting interactions between OPCs and endothelial cells, thereby accelerating bone repair in JIO. Specifically, BCA inhibits the differentiation of mature osteoclasts, expands the pool of OPCs, and stimulates PDGF-BB secretion, which in turn promotes type H vessel formation. These findings highlight the potential of BCA as a promising therapeutic candidate for the treatment of LCPD. Future studies should focus on optimizing the dosage of BCA and developing strategies to improve its solubility and bioavailability to further enhance its clinical potential.

The mechanisms by which bioactive metabolites regulate type H vessels for osteonecrosis of the femoral head treatment are summarized in Table 5.

**TABLE 5:** Therapeutic mechanisms of bioactive metabolites targeting type H vessels in osteonecrosis of the femoral head treatment.

Metabolite	Sources	Dose and duration	Model and administration	Target cells	Mechanism of action	Therapeutic effects	References
Biochanin A	*Cicer arietinum* L. [Fabaceae]	5 mg/kg every other day; 5 weeks	JIO mouse model (intraperitoneal injection)	OPCs	Stimulates PDGF-BB secretion	Promote OPCs accumulation, enhance type H vessel formation and bone regeneration, thereby alleviating femoral head collapse	Huang et al. (2025b)

## 4 Therapeutic potential of bioactive metabolites in other skeletal disorders *via* type H vessels

As discussed in previous sections, bioactive metabolites show promising therapeutic effects in major skeletal disorders through regulation of type H vessels. Importantly, this therapeutic potential extends to other skeletal conditions where type H vessel dysfunction plays a critical role. Recent studies indicate that excessive prenatal exposure to glucocorticoids can inhibit type H vessel formation, impair osteogenesis, and ultimately lead to steroid-related skeletal diseases, including fetal bone dysplasia (Shangguan et al., 2021; Su et al., 2025). Moreover, a characteristic reduction in type H vessel abundance has been observed in osteopenia of the subchondral bone of the mandibular condyle induced by botulinum toxin, demonstrating the universal regulatory role of type H vasculature in bone homeostasis across different skeletal sites (Li H. et al., 2021). However, current therapeutic strategies for these conditions lack research on targeted modulation of type H vessels using bioactive metabolites, which represents a promising new research direction.

Beyond direct hormonal exposure, prenatal dexamethasone exposure (PDE) has been shown to impair offspring skeletal development. Shangguan et al. revealed that PDE activates the GR/CEBPα/miR-34c axis to suppress PDGFRβ/FAK pathway activity, resulting in type H vessel deficiency and skeletal dysplasia (Shangguan et al., 2021). Similarly, Chai et al. found that prenatal corticosteroid treatment in mice downregulates Ezh2 expression in osteoclast precursors, reducing PDGF-BB secretion and subsequent type H vessel formation, ultimately leading to inadequate bone mineralization (Chai et al., 2020). These studies collectively demonstrate that glucocorticoids disrupt fetal bone metabolism through type H vessel inhibition. Given the established capacity of bioactive metabolites to modulate PDGF-BB secretion from OPCs and promote type H vessel formation, targeting these pathways with bioactive metabolites represents a promising therapeutic approach for hormone-induced vascular-related bone disorders.

From a perspective of skeletal development, both the mandibular condyle and long bones undergo endochondral ossification, suggesting shared vascular regulatory mechanisms. Type H vessels play crucial roles not only in long bone metabolism but also in the maintenance of mandibular bone homeostasis. Li et al. provided the first definitive evidence of type H vessels in murine mandibular bone, demonstrating their similar age-dependent decline (Li H. et al., 2021). Using a botulinum toxin-induced mandibular osteopenia model, the team observed that a significant reduction in type H vessel density correlated with pronounced bone loss. Further studies have revealed that deferoxamine mesylate effectively reverses bone loss by promoting type H endothelial cell proliferation through activation of the HIF-1α signaling pathway. This discovery not only identifies novel therapeutic targets for mandibular bone formation and repair but also, more importantly, reveals that bone disorders at different anatomical sites may share common vascular regulatory mechanisms from a vascular biology perspective. These findings provide a theoretical basis for developing bioactive metabolites targeting type H vessels to treat other potential skeletal disorders.

## 5 Summary and perspectives

Since their first description 11 years ago, type H vessels have been identified as a distinct vascular subtype within bone and are already recognized as a key indicator of bone formation and bone mass (Kusumbe et al., 2014; Wang L. et al., 2017). Extensive studies have since investigated the role of type H vessels in skeletal disorders and explored various strategies to modulate their abundance (Peng et al., 2020; Liu X. et al., 2023). Current evidence indicates that type H vessels exert beneficial effects in osteoporosis, bone defects, fractures, and osteonecrosis of the femoral head, where increased vessel abundance enhances osteogenesis and accelerates tissue repair. In contrast, excessive formation of type H vessels in the subchondral bone contributes to osteophyte growth and cartilage degeneration in osteoarthritis, acting as a pathogenic factor that requires suppression.

A growing number of therapeutic approaches, including modern pharmacological agents (Hu Y. et al., 2020; Ruan et al., 2023; Xu et al., 2023), traditional medicines (Li et al., 2020; Lu et al., 2022; Tian et al., 2022), physical and exercise therapies (Bai et al., 2021; Wang et al., 2022; Huang L. et al., 2025), and tissue engineering techniques (Yan et al., 2024; Li J. et al., 2025), are being explored to regulate type H vessels. Among these, plant-derived bioactive metabolites from traditional Chinese medicine represent a promising direction due to their natural abundance and diverse pharmacological activities. These metabolites have demonstrated significant potential in modulating angiogenesis and osteogenesis coupling through the regulation of type H vessels. The anti-osteopathic mechanisms of these metabolites can be classified according to their molecular mechanisms. Harmine, nuciferine, aucubin, acacetin, astragaloside IV, and biochanin A promote the formation of type H vessels by stimulating PDGF-BB secretion from OPCs. Curcumin, Panax quinquefolium saponin, ginsenoside Rg1, tetramethylpyrazine, ophiopogonin D, osthole, and icariin promote type H vessel formation by enhancing VEGF expression. In contrast, soybean isoflavones, isoliquiritigenin, and resveratrol inhibit VEGF-related signaling pathways, thereby suppressing abnormal type H vessel formation. Given that several upstream regulators of type H vessels have been identified in recent years, the development of additional bioactive metabolites targeting these distinct mechanisms may open new avenues for the treatment of bone disorders.

Despite the promising therapeutic potential of targeting type H vessels with bioactive metabolites, several limitations remain to be addressed. First, many plant-derived metabolites suffer from poor bioavailability due to limited water solubility, which compromises their absorption, systemic distribution, and overall pharmacological efficacy. In addition, *in vivo* studies often test only one selected dose, and more systematic dose-ranging experiments would be helpful to clarify the dose–response relationship and determine the optimal effective dose. Moreover, comprehensive pharmacokinetic studies and long-term safety evaluations are often lacking, hindering a full understanding of their therapeutic window and potential adverse effects. Second, some of these metabolites are recognized as pan-assay interfering substances (Magalhães et al., 2021), which may lead to false-positive results in high-throughput or *in vitro* screening. Therefore, careful integration of *in vitro* and *in vivo* evidence is required to accurately determine their specific targets and biological relevance. Third, the current body of research remains largely preclinical, with no clinical trials yet validating the efficacy or safety of these compounds in humans. Clinical investigations are essential to confirm their pharmacodynamics, optimal dosing, and therapeutic potential, serving as a critical step toward translating natural bioactive compounds from experimental models into viable interventions for skeletal disorders.

To further harness the therapeutic potential of bioactive metabolites targeting type H vessels, future research should focus on several key directions. First, enhancing the bioavailability, pharmacokinetic properties, and safety profiles of these compounds is essential, which can be achieved through structural optimization, novel drug delivery systems, or formulation improvements (Rahman et al., 2020). For example, coupling bioactive metabolites with nanomaterials may improve their *in vivo* bioavailability and targeting specificity (Huang et al., 2024), while compounds with potential neurotoxicity, such as harmine, can be reformulated into emulsions to enhance biosafety (Huang et al., 2018). Second, the synergistic effects between natural products or with other therapeutic approaches should be explored, as such integrated strategies may not only enhance efficacy but also reduce adverse effects. Third, expanding investigations to other type H vessel–related skeletal disorders, including mandibular osteonecrosis (Guo et al., 2025), bone tumors (Yip et al., 2021), spondyloarthritis (Kaaij et al., 2023), and bone development disorder (He et al., 2021), may reveal broader applications of type H vessel modulation. In addition, assessing the effects of bioactive metabolites on other specialized vascular subtypes in bone, such as type R and type S vessels, and comparing their regulatory differences with type H vessels, could provide novel mechanistic insights. Finally, integrating multi-omics approaches, advanced imaging techniques, and tissue engineering methods will facilitate a comprehensive understanding at cellular and molecular levels of how these metabolites regulate angiogenesis–osteogenesis coupling and exert their therapeutic effects. Overall, these efforts are expected to bridge the gap between preclinical research and clinical application, providing a foundation for the development of bioactive metabolite-based therapies targeting type H vessels, thereby enabling safe, effective, and precise interventions in orthopedics.
